# Supplementary figures and images for: A chemosensory-like histidine kinase is dispensable for chemotaxis in vitro but regulates the virulence of Borrelia burgdorferi through modulating the stability of RpoS
Source: PLoS Pathog. 2023 Nov 27;19(11):e1011752. doi: 10.1371/journal.ppat.1011752 (PMC10703414; doi:10.1371/journal.ppat.1011752)

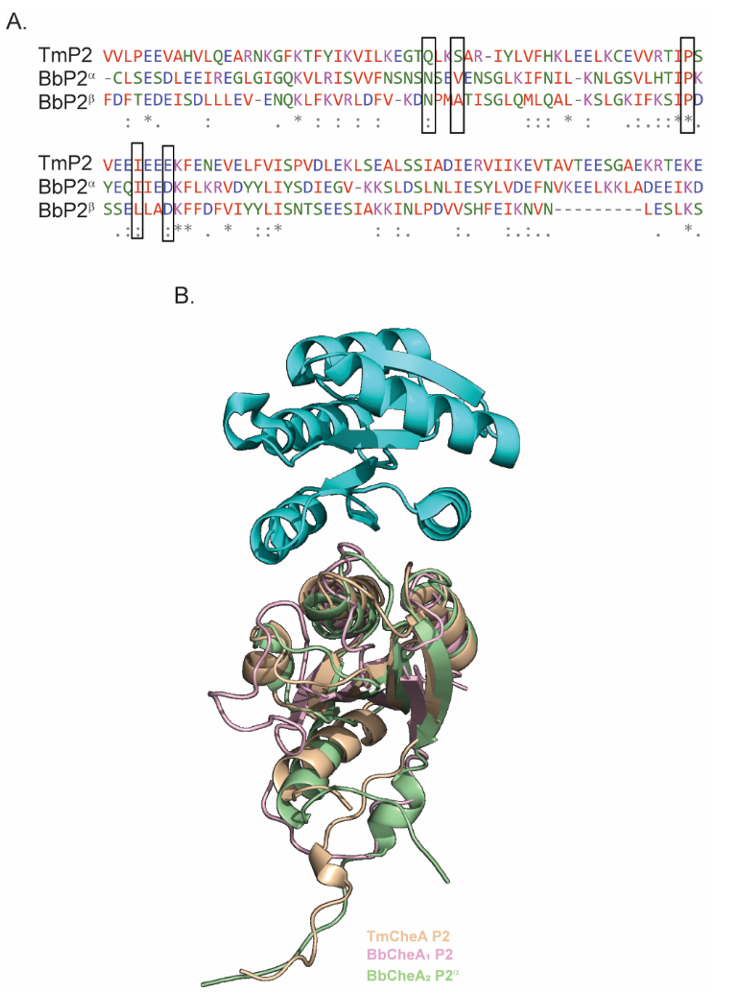

Supplement: S1 Fig — (A) Sequence alignment of BbCheA2 P2α and P2β domains compared to TmCheA P2 domain. Conserved residues at the CheY:P2 domain binding site interface are indicated with black boxes. (B) Structural superimposition of BbCheA1 P2 (pink) and BbCheA2 P2α (green) domains with TmCheA P2:CheY complex (tan, PDB:1U0S) [5]. Multiple sequence alignment analysis (MSA) were generated using Clustal Omega [6] and figures were prepared in PyMol [7]. (TIFF) [file ppat.1011752.s001.tiff]

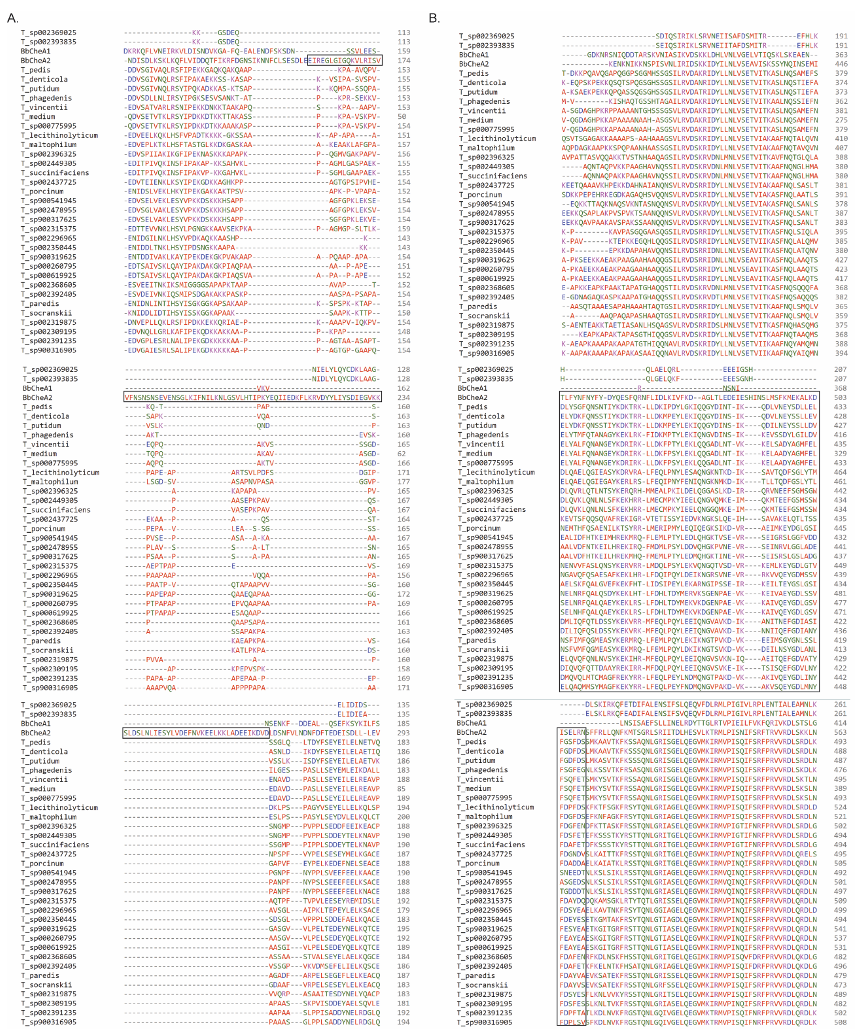

Supplement: S2 Fig — (A) MSA of Treponema spp. CheA P2 domain sequences. BbCheA1 and BbCheA2 sequences are included for comparison. The location of BbCheA2 P2α is marked with a black box. (B) MSA of Treponema spp. CheA P3 domain sequences. BbCheA1 and BbCheA2 sequences are included for comparison. The location of BbCheA2 P3 domain is marked with a black box. Sequences collected using Annotree [1], MSA files generated using Clustal Omega [6]. (TIFF) [file ppat.1011752.s002.tiff]

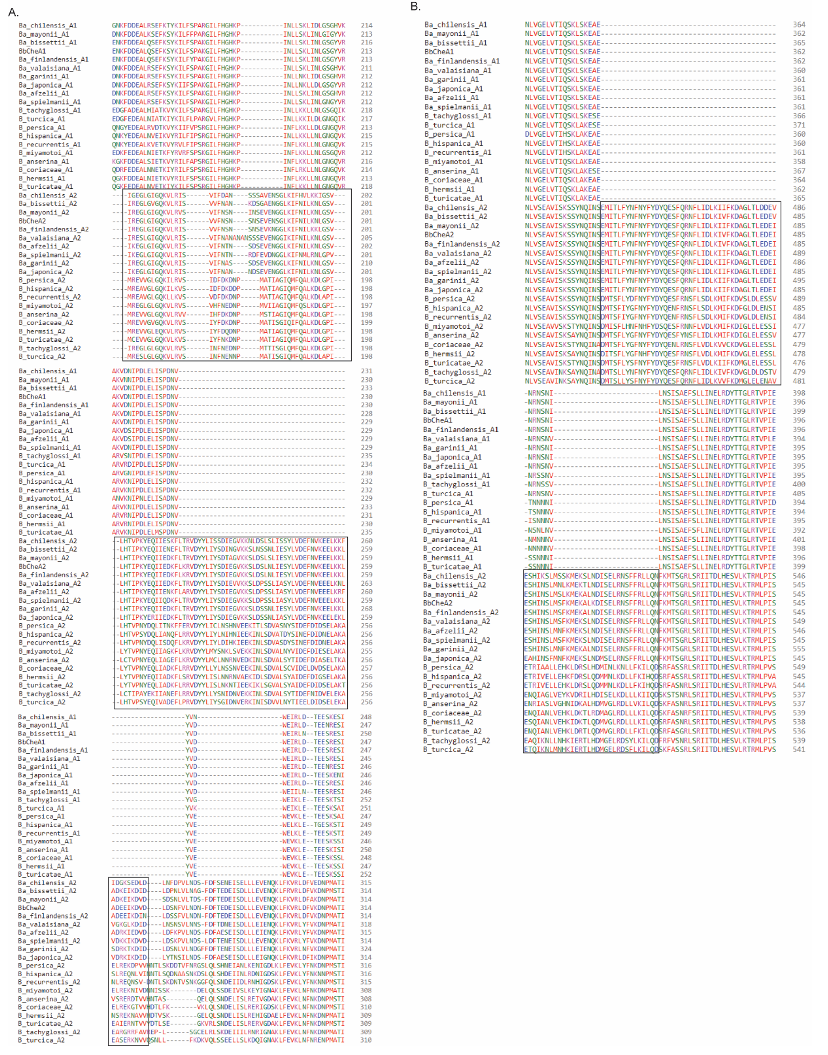

Supplement: S3 Fig — (A) MSA of Borrelia (B_) and Borreliella (Ba_) spp. CheA1 and CheA2 P2 domain sequences. Location of BbCheA P2α marked with black box. (B) MSA of Borrelia (B_) and Borreliella (Ba_) spp. CheA1 and CheA2 P3 domain sequences. Location of BbCheA2 extended P3 domain sequence marking with black box. Sequences collected using Annotree [1], MSA files generated using Clustal Omega [6]. (TIFF) [file ppat.1011752.s003.tiff]

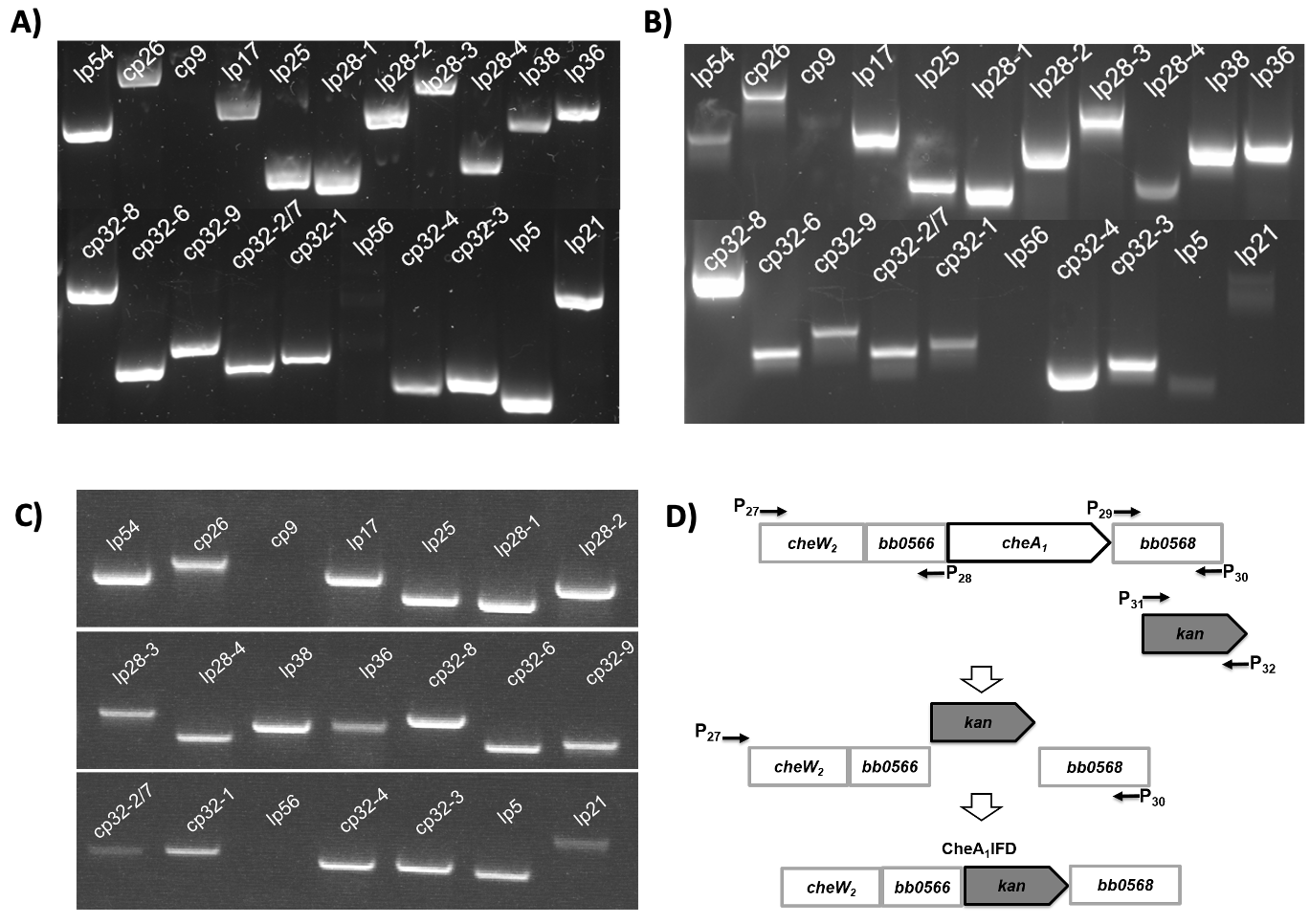

Supplement: S4 Fig — PCR was used to detect the plasmid profile of WT (A), cheA1mut (B) and cheA1com (C). The primers used were described previously [8]. (D) A diagram illustrating the construction of CheA1IFD. To construct an in frame deletion mutant, primer pair P27/P28 and P29/P30 were used to amplify the upstream and downstream flanking region of cheA1. Primer pair P31/P32 was used to amplify a promoterless kanamycin cassette (kan). cheA1 was in-frame replaced by kan via PCR fusion technique with primer pair P27/P30. The resulting PCR fusion amplicon was cloned into pJet1.2 vector forming CheA1IFD. (TIFF) [file ppat.1011752.s004.tiff]

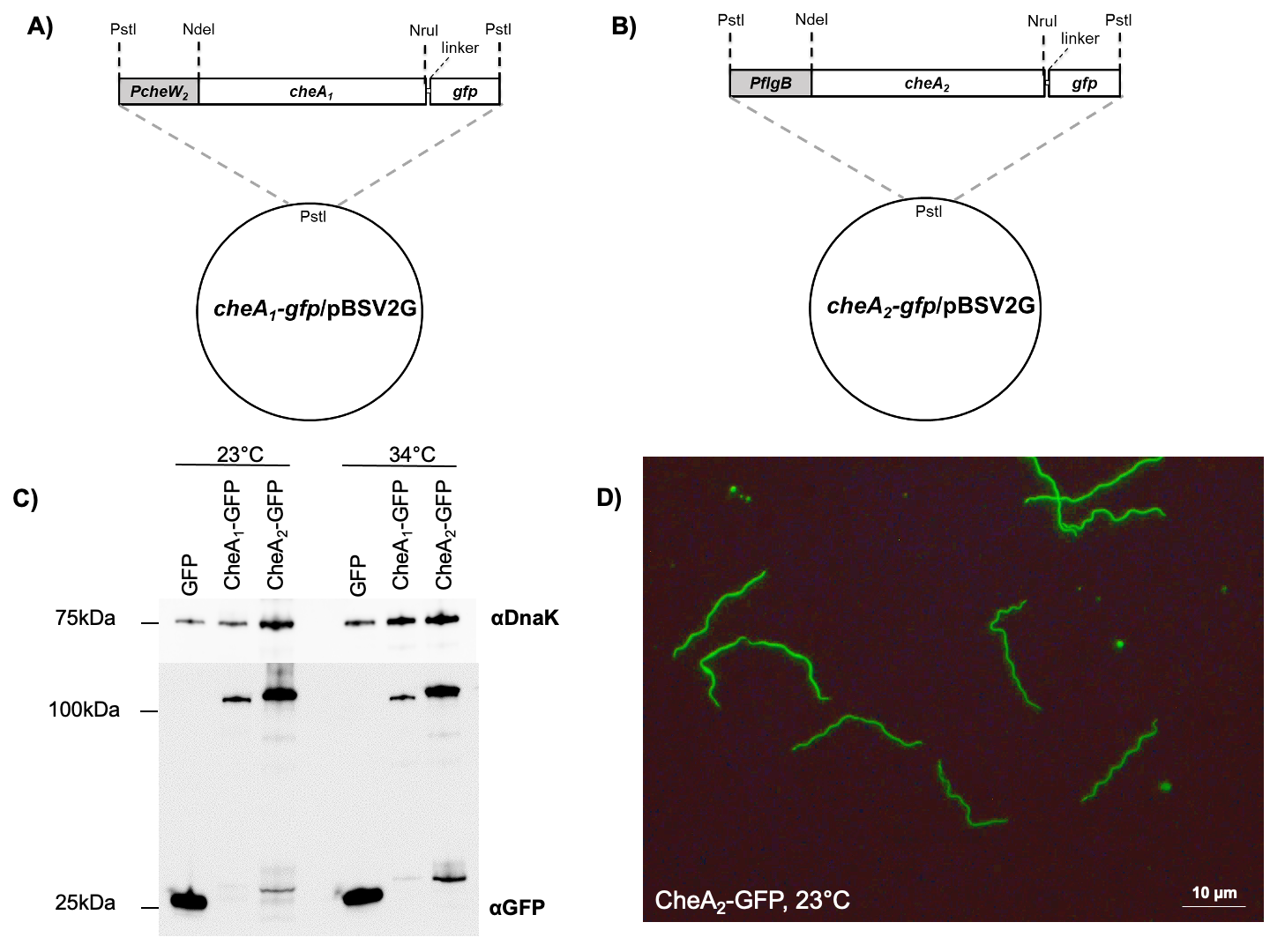

Supplement: S5 Fig — (A) Construction of cheA1-gfp/pBSV2G. The upstream flanking region (PcheW2, 417 bp) of cheW2 gene was PCR amplified and fused to cheA1 using the engineered restriction sites as shown. The fused PcheW2-cheA1 fragment was then fused to gfp gene containing a 5 x Gly linker at the indicated restriction sites and cloned into pBSV2G, a shuttle vector of B. burgdorferi [3], yielding cheA1-gfp/pBSV2G. (B) Construction of cheA2-gfp/pBSV6G. Similarly, the flgB promoter [4] was PCR amplified and fused to cheA2 gene followed by fusion to gfp with 5 x Gly linker at the indicated restriction sites prior to cloning into pBSV2G. The obtained construct was used to complement a previously constructed cheA2 mutant strain [9]. (C) Immunoblot analysis of GFP fusion proteins. Cell lysates from B. burgdorferi strains expressing GFP, CheA1-GFP, or CheA2-GFP cultured at UF conditions (23°C/pH 7.6) or routine laboratory cultural condition (34°C/pH 7.6) were analyzed on SDS-PAGE and probed with antibodies against GFP or DnaK (as loading control). No excessive degradation of GFP fusion proteins was observed under both culture conditions. (D) CheA2-GFP does not have a polar localization like CheA1-GFP. B. burgdorferi strain carrying CheA2-GFP construct was cultivated under UF tick condition. CheA2-GFP appeared diffused with no specific cellular localization observed. (TIFF) [file ppat.1011752.s005.tiff]

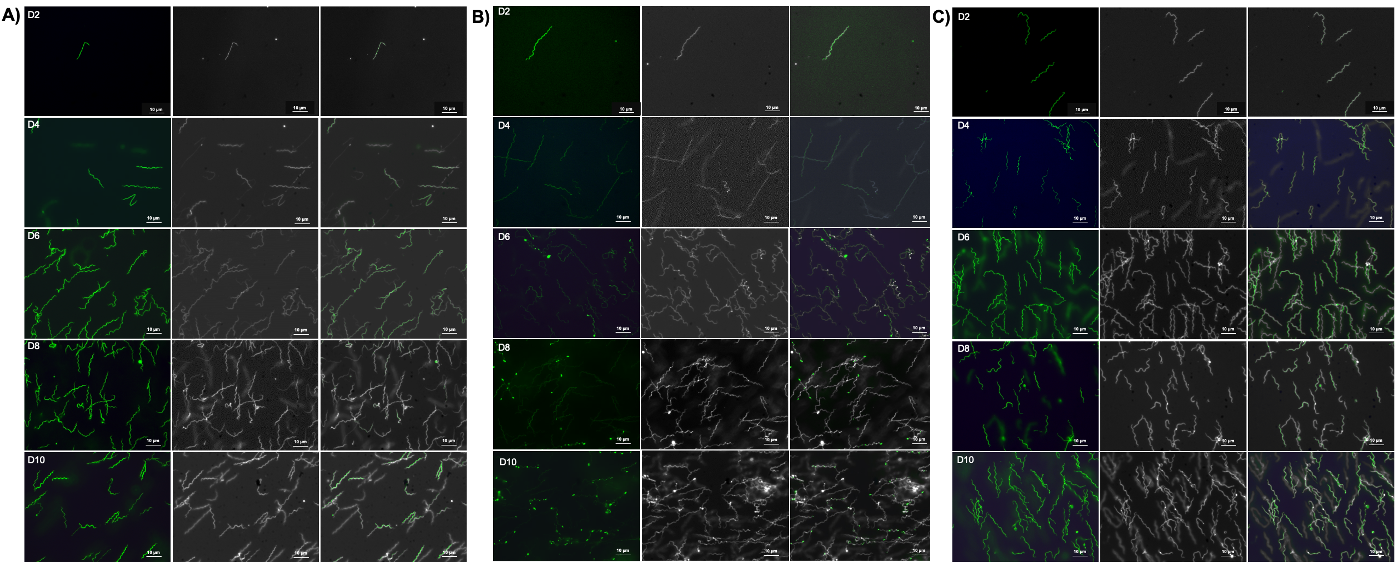

Supplement: S6 Fig — 105 cells/ml of B. burgdorferi strains that express (A) GFP, (B) CheA1-GFP, or (C) CheA2-GFP were inoculated into 10 ml fresh BSK-II medium and cultivated at 34°C/pH 7.6. Images were taken every two days at ×200 magnification using a Zeiss Axiostar Plus microscope. Scale bars represent 10 μm. (TIFF) [file ppat.1011752.s006.tiff]

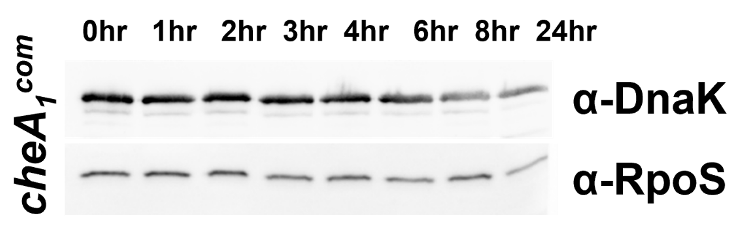

Supplement: S7 Fig — The stability of RpoS protein was examined in stationary phase of B31 A3-68 cheA1com upon protein synthesis arrest with spectinomycin. Samples were harvested at the indicated time points and probed using antibody against RpoS and DnaK (as a loading control). (TIFF) [file ppat.1011752.s007.tiff]

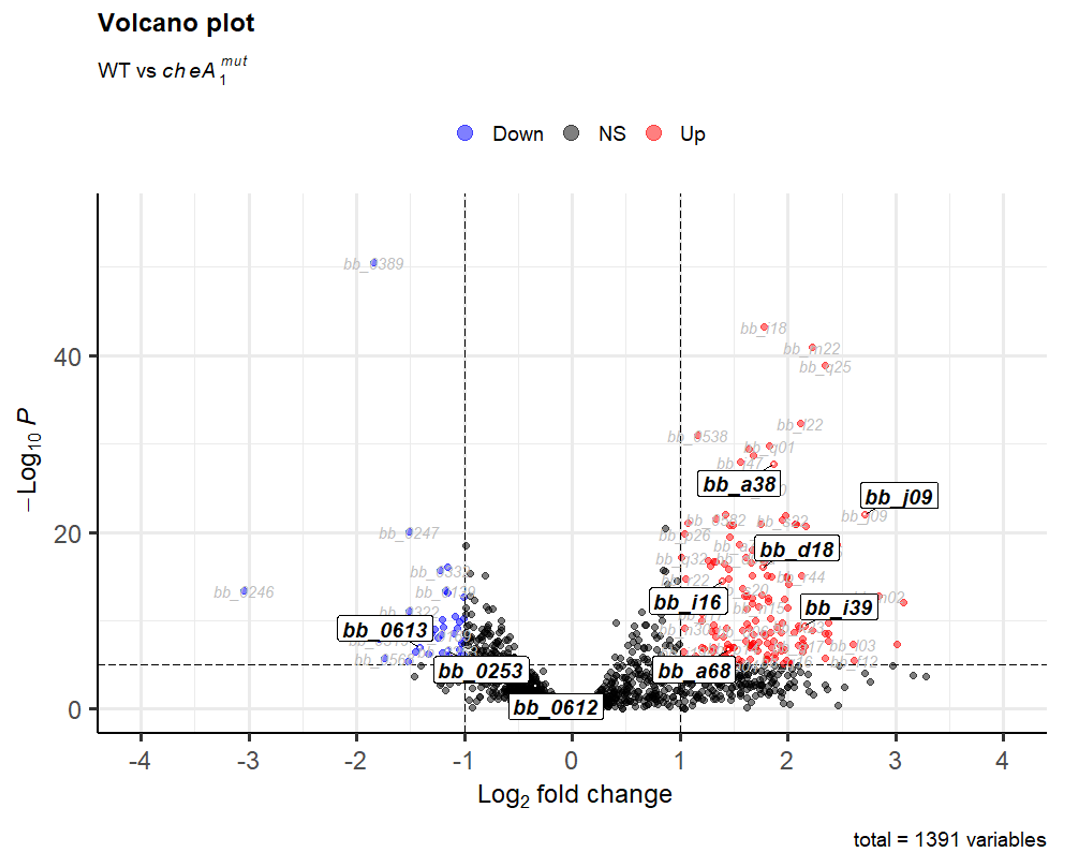

Supplement: S8 Fig — A total of 1391 DEG between WT and mutant were plotted. Three down-regulated protease genes (bb0613, bb0612, and bb0253) and six up-regulated RpoS-repressed genes (bb_a38, bb_a68, bb_d18, bb_i16, bb_i39, bb_j09) in cheA1mut were highlighted in the volcano plot. (TIFF) [file ppat.1011752.s008.tiff]
