# Supplementary material for: A chemosensory-like histidine kinase is dispensable for chemotaxis in vitro but regulates the virulence of Borrelia burgdorferi through modulating the stability of RpoS
Source: PLoS Pathog. 2023 Nov 27;19(11):e1011752. doi: 10.1371/journal.ppat.1011752 (PMC10703414; doi:10.1371/journal.ppat.1011752)
Supplement: S1 Table — (PDF) [file ppat.1011752.s009.pdf]

Supplementary data

A chemosensory-like histidine kinase is dispensable for chemotaxis *in vitro* but harnesses the virulence of *Borrelia burgdorferi* through modulating the stability of RpoS

Ching Wooen Sze[1], Kai Zhang[1], Michael J. Lynch[2], Radha Iyer[3] , Brian R. Crane[2], Ira Schwartz[3], and Chunhao Li[1,4*]

Department of Oral Craniofacial Molecular Biology, Virginia Commonwealth University, Richmond, Virginia, 23298[1]

Department of Chemistry and Chemical Biology, Cornell University, Ithaca, New York, 14853[2]

Department of Microbiology and Immunology, New York Medical College, Valhalla, New York, 10595[3]

Department of Microbiology and Immunology, Virginia Commonwealth University, Richmond, Virginia, 2329[4]

Running title: *Borrelia burgdorferi* chemotaxis histidine kinase and virulence

Key words (Lyme disease /*Borrelia burgdorferi*/ Chemotaxis histidine kinase/ Virulence/RpoS)

[*]*Corresponding author. Mailing address: Chunhao Li, 1112 E. Clay Street, Health Science Research Building Room 105, Richmond, Virginia 23298. Tel: (804) 628-4401; Email: cli5@vcu.edu.*

Material and Methods

Multiple sequence alignment analysis (MSA) of spirochete CheA. MSA analysis was performed on CheA sequences for spirochetes whose genomes have been sequenced and annotated. Sequences were retrieved using Annotree (1) with the following search parameters: KEGG: K03407, percent identity: 30, E-value: 0.00001, percent subject alignment: 70, percent query alignment: 70. Sequences were obtained for all bacterial species where only spirochetes were analyzed. Within the spirochete CheA sequences, individual sequences were filtered and redundant sequences (both in sequence and species) were removed. From this list, the number of CheA isoforms per species were calculated and segregated into groups according to the total number of CheA isoforms per species. From the single CheA isoform group, we chose to analyze only the members from the order *Treponematales* (34 total sequences). For the two CheA isoform group, we chose to analyze only those members from the family *Borreliaceae* (i.e. *Borrelia* spp. and *Borreliella* spp.), totaling 40 individual unique sequences (i.e. 20 $CheA_1$ and 20 $CheA_2$). Fasta files were fed into Clutal Omega7 using *B. burgdorferi* $CheA_1$ and $CheA_2$ as query sequences.

Construction of GFP reporter plasmids for the localization of $CheA_1$ and $CheA_2$ in *B. burgdorferi.* For the localization of $CheA_1$, primer pair P_{41}/P_{42} was used to amplify 417 bp upstream of *cheW*$_2$ encompassing the putative promoter region of the *cheW*$_2$ operon (2) with engineered PstI and NdeI cut site at the 5' and 3' end, respectively; primer pair P_{43}/P_{44} for *cheA*$_1$ gene with engineered NdeI and NruI cut site at the 5' and 3' end, respectively; and lastly primer pair P_{45}/P_{46} for the amplification of *gfp* gene containing a 5 x Gly linker, with engineered NruI and PstI cut site, respectively. The obtained 3 amplicons were cloned into pGEM-T-easy vector and step wise ligation using the engineered restriction sites was performed to obtain the full fusion product *PW*$_2$*-cheA*$_1$*-gfp* which was then cloned into pBSV2G vector at the PstI cut site (3), yielding *cheA*$_1$*-gfp*/pBSV2G plasmid (**Fig. S6A**). Similarly, for *cheA*$_2$*-gfp*/pBSV2G construct, *flgB* promoter (4) was PCR amplified using primer pair P_{47}/P_{48} and fused

to *cheA₂ orf* amplified with primer pair P_{49}/P_{50} followed by fusion to *gfp* gene using the same restriction sites as *cheA₁-gfp*/pBSV2G. All primers used are listed in Table S1.

RNA isolation using Qiagen RNeasy mini kit. To prepare RNA for RNA-seq, 50 ml of mid-log phase *B. burgdorferi* cells cultured at 34°C, pH 7.4 were harvested and RNA was extracted using RNeasy mini kit following manufacturer's instruction. Briefly, bacterial pellet was resuspended in 700 µl Buffer RLT containing 1 % β-mercaptoethanol and vortexed vigorously for 5–10 seconds. Suspension was transferred to cell homogenizer tube and centrifuged at maximum speed for 1 minute. Equal volume of 70% ethanol was added to the flow through and mixed well by pipetting. The suspension was transferred to RNeasy Mini spin column placed in a 2 ml collection tube and centrifuged for 15 seconds at ≥ 8,000 x *g*. 350 µl of Buffer RW1 was added to the RNeasy spin column, and centrifuged for 15 seconds at ≥ 8,000 x *g* to wash the spin column membrane followed by on-column DNase I digestion at room temperature for 15 minutes. 350 µl of Buffer RW1 was then added to the RNeasy spin column, incubated for 5 minutes followed by centrifugation for 15 seconds at ≥ 8,000 x *g*. The RNeasy Mini spin column was washed twice with 500 µl of Buffer RW2 before elution using 50 µl RNase-free water. RNA concentration was quantified using a NanoDrop and stored in -80°C freezer before subjecting to RNA-seq analysis.

RNA-seq analysis. Extracted RNA was subjected to ribosomal depletion prior to sequencing run using Illumina MiSeq Reagent kit v3 for 600 cycles. Reads were mapped to *Borrelia burgdorferi* genome (GCA_000008685.2_ASM868v2) using Bowtie2 software. Genes that were differentially expressed between the wild type and mutant cells were determined using DEseq2 pipeline with the Benjamini-Hochberg (BH) adjustment to calculate for each gene an adjusted *p* (*p*adj) to determine the fraction of false positive rate (FDR) among them. Differentially expressed genes identified using a 5% FDR (*p*adj < 0.05) and a two-fold expression difference were considered to identify genes that are differentially

regulated in the mutant relative to the wild type. Significantly differentially expressed genes (DEG) were then plotted on a Volcano plot.

Table S1. Oligonucleotide primers used in this study [a]

Primer	Description	Sequences
**P_{41}**	$CheA_1$ localization, *$cheW_2p$* (F)	5'- CTGCAGAATGCTGAATTCTATTGCAG -3'
**P_{42}**	$CheA_1$ localization, *$cheW_2p$* (R)	5'- CATATGGAATGTAATTTAAATCGC -3'
**P_{43}**	$CheA_1$ localization, *$cheA_1$* (F)	5'-CATATGGATAGTAGTGATGTTAT-3'
**P_{44}**	$CheA_1$ localization, *$cheA_1$* (R)	5'-TCGCGATTTTATAAGTTTAGTTA-3'
**P_{45}**	$CheA_1$ localization, *gfp* (with linker) (F)	5'- TCGCGAAAAGGTGGAGGTGGAGGTAAGAAGGAG ATATACATATGAG -3'
**P_{46}**	$CheA_1$ localization, *gfp* (R)	5'- CTGCAGTTAGTATAGTTCATCCATGCCATG -3'
**P_{47}**	$CheA_2$ localization, *flgBp* (F)	5'- CTGCAGTAATACCCGAGCTTCAAG -3'
**P_{48}**	$CheA_2$ localization, *flgBp* (R)	5'- CATATGACCTCCCTCATTTAAAATTG -3'
**P_{49}**	$CheA_2$ localization, *$cheA_2$* (F)	5'-CATATGGAAATATTAGATTTGG-3'
**P_{50}**	$CheA_2$ localization, *$cheA_2$* (R)	5'-TCGCGACTCCTTAGTATCCTTTTG-3'

[a] The underlined sequences are the engineered restriction cut sites for DNA cloning; F, forward; R, reverse.

Reference

1. Mendler K, Chen H, Parks DH, Lobb B, Hug LA, Doxey AC. AnnoTree: visualization and exploration of a functionally annotated microbial tree of life. Nucleic Acids Res. 2019;47(9):4442-8.
2. Sze CW, Li C. Chemotaxis Coupling Protein CheW(2) Is Not Required for the Chemotaxis but Contributes to the Full Pathogenicity of Borreliella burgdorferi. Infect Immun. 2023;91(4):e0000823.
3. Elias AF, Bono JL, Kupko JJ, 3rd, Stewart PE, Krum JG, Rosa PA. New antibiotic resistance cassettes suitable for genetic studies in Borrelia burgdorferi. J Mol Microbiol Biotechnol. 2003;6(1):29-40.
4. Ge Y, Charon NW. Molecular characterization of a flagellar/chemotaxis operon in the spirochete Borrelia burgdorferi. FEMS Microbiol Lett. 1997;153(2):425-31.
5. Park SY, Beel BD, Simon MI, Bilwes AM, Crane BR. In different organisms, the mode of interaction between two signaling proteins is not necessarily conserved. Proc Natl Acad Sci U S A. 2004;101(32):11646-51.
6. Sievers F, Wilm A, Dineen D, Gibson TJ, Karplus K, Li W, et al. Fast, scalable generation of high-quality protein multiple sequence alignments using Clustal Omega. Mol Syst Biol. 2011;7:539.

7. The PyMOL Molecular Graphics System. 2.0 ed: Schrödinger, LLC.
8. Elias AF, Stewart PE, Grimm D, Caimano MJ, Eggers CH, Tilly K, et al. Clonal polymorphism of Borrelia burgdorferi strain B31 MI: implications for mutagenesis in an infectious strain background. Infect Immun. 2002;70(4):2139-50.
9. Li C, Bakker RG, Motaleb MA, Sartakova ML, Cabello FC, Charon NW. Asymmetrical flagellar rotation in Borrelia burgdorferi nonchemotactic mutants. Proc Natl Acad Sci U S A. 2002;99(9):6169-74.
