# Supplementary material for: A chemosensory-like histidine kinase is dispensable for chemotaxis in vitro but regulates the virulence of Borrelia burgdorferi through modulating the stability of RpoS
Source: PLoS Pathog. 2023 Nov 27;19(11):e1011752. doi: 10.1371/journal.ppat.1011752 (PMC10703414; doi:10.1371/journal.ppat.1011752)
Supplement: Attachment — Submitted filename: Point-by-point responses-D-23-01340-Final.docx [file ppat.1011752.s012.docx]

We thank three Reviewers for their overall enthusiasm and constructive comments and suggestions. We revised this manuscript accordingly. Below are our point-by-point responses to these comments.

**Part I – Summary**

**Reviewer #1:**

*1. CheA1, chemotaxis, tick transmission, and dissemination in the mouse. Throughout the manuscript the authors maintain that CheA1 is critical for tick transmission. Since this almost certainly involves chemotaxis, it is not clear why they claim, based solely on in vitro data, that CheA1 is not required for chemotaxis. As they note towards the end of the Discussion in their 2017 CheY2 paper, one cannot consider in vitro studies to be definitive with respect to chemotaxis in vivo. Moreover, given that CheY2 is believed to be the cognate response regulator for CheA1, a closer comparison of the CheA1 and CheY2 phenotypes (which seem quite similar, length of duration at the injection site not withstanding) seems worthwhile. In both cases, this reviewer suggests describing CheA1 and CheY2 as dispensable for chemotaxis in vitro. I note parenthetically that the statement on p25 that CheA1 has no known effector seems puzzling given published work with CheY2. The authors appear to believe that the dissemination and survival defect of the cheA1 mutant in mice is due to diminished expression of RpoS-dependent gene products. The data, as presented, however, do not eliminate the possibility that CheA1, which is known to be RpoS-dependent, is involved in chemotaxis within the mammal.*

**Response:** We appreciate this thoughtful comment and fully agree that our current results do not rule out the possibility that CheA_1_ is required for chemotaxis in vivo, e.g., during tick transmission. In fact, we discussed this possibility on pages 21 and 22 and in our previous publications^1,2^. On pages 25 and 26 we further entertain the possibility that CheA_1_ contributes to phases of host dissemination, especially in consideration of the available data on CheW_2_ and CheY_2_. Nonetheless, additional studies will be needed to resolve this complex issue. To that end, we recently constructed a CheA_1_ mutant that ectopically expresses GFP and are planning to monitor its swimming behavior in mice and ticks using intravital imaging and confocal microscopy, as previously reported^3-5^. This study will allow us to decipher the role of CheA_1_ in chemotaxis in vivo. However, due to our lack of experience with such approaches, we will carry out these experiments in collaborative future studies.

As to whether or not CheY_2_ is an effector of CheA_1_, under in vitro conditions, both CheA_1_ and CheA_2_ are capable of phosphorylating all three CheYs but whether this occur in vivo remains unclear^6^. The conjecture that CheY_2_ is the cognate response regulator for CheA_1_ is solely based on its gene location. In addition, we noted differences in the infectivity pattern between *cheA_1_* and *cheY_2_* mutants as presented in our discussion. Given that CheA_1_ is a kinase whose effect typically acts through its phosphorylation of a response regulator, if CheY_2_ is truly the cognate response regulator of CheA_1_, one would expect the two mutants to phenocopy each other, just like the *cheA_2_* and *cheY_3_* mutants^2,7^. The infection study of the *cheY_2_* mutant showed that the mutant failed to establish localized infection and did not multiply at the injection site^8^, which is in stark contrast to our observations of the *cheA_1_* mutant, as shown in Fig 5. Notably, CheY_2_ is not a DNA binding protein. Even if it is an effector of CheA_1_, it cannot directly control gene expression; thus, CheA_1_ must act through other transcriptional regulators. While we do not reject the possibility of CheY_2_ being a response regulator for CheA_1_, the differences in phenotype between *cheA_1_* and *cheY_2_* mutants led us to conclude that CheA_1_ may have an alternative response regulator which we speculated to be BB0566, a putative anti-sigma or anti-anti-sigma factor. Nevertheless, we have revised our manuscript to address these two concerns as suggested.

*2. The comparison of CheA1 and CheA2 structural models with the solved structure of Thermotoga maritime CheA is very interesting and leads to some testable hypotheses to explain functional differences between CheA1 and CheA2. However, the rationale provided for this first section of the paper – that CheA1 is dispensable for chemotaxis – is perplexing for two reasons. One is that, per above, the dispensability appears to be for chemotaxis in vitro. The second is that no data for CheA1 have been presented yet in the manuscript or published previously to provide context for the statement. The greater structural similarity between CheA1 and other bacterial CheAs further implies some involvement of CheA in chemotaxis even though it is less ‘spirochete-like’.*

**Response:** Based on our data obtained using capillary tube assay and swimming plate analysis as shown in Fig 4 along with our previous publication^9^, we concluded that CheA_1_ is dispensable for chemotaxis in vitro as the mutant is still chemotactic toward N-acetyl-glucosamine, a defined chemoattractant of *B. burgdorferi*. In addition, swimming plate analysis showed the presence of a swim ring with a comparable diameter as the wild type, indicating that the mutant is chemotactic and able to migrate toward nutrients. We have revised the statement to “CheA_1_ is dispensable for chemotaxis in vitro” as suggested. We agree with the reviewer that CheA_1_ may potentially be involved in chemotaxis in vivo as speculated in our discussion, page 21.

*3. The authors present data that CheA1 and CheA2 are expressed constitutively under the in vitro conditions studied herein. However, they also note and seem to rely upon previous microarray studies by Iyer et al. showing that CheA1 and CheA2 are differentially expressed with divergent expression profiles in ticks and mammals.*

**Response:** Our intention here was to show that CheA_1_ and CheA_2_ proteins can be detected under the in vitro conditions specified, and not to emphasize the differential expression of CheA_1_ and CheA_2_. To address this concern, we removed supplementary figure 5 accordingly.

*4. Discussion page 25. The evidence presented shows that CheA1 controls expression of the alternative sigma factor RpoS at the protein level through a previously unrecognized role regulating transcription of the Clp proteolytic cascade. This unanticipated finding results from RNAseq analysis showing global transcriptional dysregulation in cheA1 mutant spirochetes. If there is less RpoS, it stands to reason that there would be more RNA polymerase with sigma70 with the resultant global effects. Consequently, the need to invoke phosphorylation of anti-sigma factors and sigma factor replacement is not apparent, not to mention the fact that, to the best of this reviewer’s knowledge, anti-sigma factors have not been described in B. burgdorferi (the proteolytic pathway for degradation of RpoS is not an anti-sigma factor pathway). Admittedly, tying all these complex pathways together mechanistically presents quite a challenge. In this reviewer’s opinion, the authors shouldn’t feel compelled to mechanistically explain all of their observations.*

**Response:** We agree and have revised the discussion accordingly. The speculation of a sigma switching theory has been removed.

*5. The authors are to be commended for going outside their comfort zone to characterize host transcriptional responses at sites inoculated with the cheA1 mutant. That the mutant gives rise to a global dampening of the local inflammatory response is another unexpected finding. However, the comments about IL-10 knockout mice at the end of the discussion are not exactly on point. IL-10 is an anti-inflammatory cytokine. In IL-10 knockouts, the inflammatory response is heightened, not diminished as shown here.*

**Response:** We agree and have revised accordingly.

**Reviewer #2:**

Minor concern:

*1. L239-251, chemotaxis experiments. Are these experiments performed at 23 ℃ or 34 ℃? Given that CheA1 showed a unique localization at 23 ℃, these experiments should be done under same conditions. Does CheA1-CheY2 crosstalk with CheA2-CheY3? Or is it possible that CheA1 phosphorylates CheY3 and then contributes to chemotaxis at a different condition?*

**Response:** These experiments were conducted at 34℃ using a well-established protocol^9,10^. However, it is technically challenging to carry out the same experiment at 23 ℃ mainly because *B. burgdorferi* poorly swims out and forms visible rings in soft agars at this temperature.

Regarding the potential crosstalk between these two systems, we think this possibility is very unlikely based on the following facts. First, *cheA_2_* and *cheY_3_* single mutants are non-chemotactic in vitro and swim in one direction. The double mutant of *cheA_2_cheY_3_* also has the identical phenotype as the single mutations in that the cells swim in one direction and are non-chemotactic. If there is crosstalk, one would expect both *cheA_2_* and *cheY_3_* single mutants to behave like wild type or at least in part, and that *cheA_2_cheY_3_* double mutant would also be chemotactic if CheA_1_-CheY_2_ can compensate for its chemotaxis role in vitro. In addition, if crosstalk exists in vivo, the *cheA_2_* mutant would not be defective in tick transmission stage as CheA_1_ would be able to rescue the phenotype^2^. Furthermore, if CheA_1_ phosphorylates CheY_3_ in vivo, we would expect to see a similar phenotype between these two mutants. However, *cheY_3_* mutant has reduced viability after tick blood meal^7^, which was not seen in *cheA_1_* mutant (Fig. 8A).

*2. L253-272: Animal infection experiments. Only 1 out of 5 mice was positive in the injection skin site for the complemented strain. This partial complementation was also observed in distal tissues and in tick-mice transmission (Fig. 8B). What is the reason for this partial complementation? Does cheA1 co-transcribe with other genes, so that expression of neighboring genes was affected in the mutant? Has the IFD mutant been complemented and that complement showed similar or different phenotypes? Was the mutant recovered from blood?*

**Response:** Complementation of CheA_1_ mutant was particularly challenging. We have made at least three different complementation constructs encompassing various region of the *cheW_2_* operon for both *cis* and *trans* complementation and only one was able to restore the expression of CheA_1_ closest to the wild-type level, which is the construct used in our manuscript. Complement strains with too much CheA_1_ expression led to a complete inability to cause infection in mice, including at the inoculation site, further highlighting the importance of CheA_1_ in the pathogenicity of *B. burgdorferi*. Our recent publication on CheW_2_ identified a promoter upstream of *cheW_2_* gene for this operon and that *cheW_2_-bb0566-cheA_1_* are co-transcribed^1^. We later found out that in addition to this promoter, there exists an alternative promoter, most likely within *bb0566*, that can also drive the expression of CheA_1_, albeit to a much lower level. The complexity of this operon adds to the challenges in obtaining a complement strain with fully restored infectivity. To address this issue, we constructed the second mutant (*cheA_1_^IFD^*) with the entire open reading frame removed and found that the two mutants have identical phenotypes. The IFD mutant was also complemented but showed a similar partial complementation phenotype. The IFD mutant was not recovered from the blood of wild-type mice but can be recovered from the blood of SCID mice, similar to the result of *cheA_1_^mut^* as shown in Table 2.

*3. Mice RNA-seq (L775-786), Fig. 11; Fig. S8, RNA-seq. Has qRT-PCR been performed to confirm the RNA-seq data? Please add data statistical significance.*

**Response:** Yes, the mice RNA-seq data has been validated using qRT-PCR. A new figure has been added to Fig. 11C. The *p*-values are indicated by the color as shown by the legend on the right-hand corner in Fig. 11A. For Fig. 11B, the cut off threshold was |log2FC| > 1, and *p* < 0.05 as indicated in the Materials and Methods section.

*4. BBD18 has been reported to repress RpoS translation. In Fig. S8, BBD18 expression was shown to be increased in cheA1 mutant? These data should be confirmed by qRT-PCR and/or immunoblot in the mutant and complement.*

**Response:** Yes, *bb_d18* was one of the up-regulated genes in *cheA_1_* mutant (Fig. S8). We verified the level of *bb_d18* using RNA from cells cultured under host-mimic condition (see the figure on right). Consistent with the RNA-seq data, *bb_d18* was highly induced in *cheA_1_* mutant. In addition, our complement strain failed to repress the level of *bb_d18* which can explain, at least in part, why we were unable to fully restore its phenotype to wild-type level in our mice infection study. The qRT-PCR data for *bb_d18* has been added to the revised Fig. 10A.

**Editorial suggestions:**
*1. L31-33 is awkward. Please revise it.*

**Response:** Revised accordingly.

*2. L103, L105, L205, L344, L377: change “is” to “was”*

**Response:** Revised accordingly.

*3. L120: revise the sentence “why CheA1 is dispensable for chemotaxis while CheA2 is required”, as it is heretofore unknow whether CheA1 is required for chemotaxis. The role of CheA1 in chemotaxis was described later in this manuscript.*

**Response:** Our publication in 2002 titled “Asymmetrical flagellar rotation in *Borrelia burgdorferi* non-chemotactic mutants”^9^ has established that CheA_1_ is dispensable for chemotaxis in vitro while CheA_2_ is required.

*4. L204: protein cellular localization. Does addition of a GFP tag at the C-terminus of CheA1 affect protein localization and function?*

**Response:** We do not believe that the addition of a GFP tag at the C-terminus of CheA_1_ will affect its localization and function for two reasons: 1) the expression of GFP alone does not show polar localization; and 2) addition of GFP to the C-terminal of CheA_2_ does not result in polar localization (Fig. S6). If GFP itself can affect the localization of CheAs, we would expect to see a similar phenotype in CheA_2_-GFP strain too.

*5. L184: change “contain” to “contained”; L185: change “does” to “did”*

**Response:** Changed accordingly.

*6. L183-184, Fig S4: Please add the plasmid profiles of the wild-type strain and the mutant*

**Response:** Revised accordingly and new figures have been added.

*7. L188: Has gene transcription been examined under these conditions?*

**Response:** This result has been removed based on reviewer #1’s comment.

*8. L336-348, Fig. 7B, it would be more informative to present the data as ratio of viable cells treated with NHS or hiNHS to the input (106 cells).*

**Response:** Agreed and revised accordingly. Fig. 7B has been replaced with newly analyzed data.

*9. L360-374, tick-mice transmission: were spirochetes re-isolated from mice tissues like the experiments done in Table 1?*

**Response:** We did not perform culture re-isolation experiment for these tissues. Specimens were harvested solely for measuring bacterial burdens.

*10. L395. Did CheA1 mutation affect the growth under this condition (37 ℃/pH6.8)?*

**Response:** No, deletion of CheA_1_ has no significant impact on cell growth under 37°C/pH6.8 condition. This new growth curve has been added to Fig. 2C.

*11. L401-404, Fig. 9B. How many times have these experiments been repeated? Data for Fig. 9C, please add data statistical analysis.*

**Response:** The immunoblot analysis in Fig. 9B has been repeated at least 3 times using independent biological replicates. The statistical analysis of Fig. 9C is indicated by the asterisks on top of the bar graph. As suggested, new statistical analysis has been added for clarity.

*12. L423-426, Fig. 10A. Data lack appropriate statistical analysis.*

**Response:** Fig. 10A has been revised accordingly*.*

*13. L442-443. L734-736. Why the animal infection route and the bacterial dose are different from other animal experiments in this study?*

**Response:** For L442-443, 10^7^ cells were used for infection instead of 10^5^. Our initial intention was to inject the mice with high doses of bacteria seeking to re-isolate *B. burgdorferi* RNA from the injection site for dual RNA-seq analysis. Unfortunately, the read was less than 0.1% coverage of the genome of *B. burgdorferi* and majority of the RNA was from mouse as expected, leaving us with only the mouse skin RNA-seq data.

For L734-736, tail-vein injection was used instead of subcutaneous injection as we wanted to determine if bypassing the initial dissemination step from mice skin to vasculature can rescue the dissemination and colonization defect of *cheA_1_* mutant.

*14. Revise the Y-axis of Fig. 11B.*

**Response:** Agreed and revised as suggested.

15. L541. Change “finding” to “findings”.

**Response:** Changed as suggested.

*16. L634-636. The authors propose that CheA1 may regulate expression through a response regulator. This can be discussed in more detail.*

**Response:** CheA_1_ is a kinase and kinase acts through a specific response regulator which is part of a two component signaling pathway.

*17. L712. “The mutant was confirmed to have the same antigen profile as cheA1 mut”. What does this mean?*

**Response:** The protein expression detected in Fig. 9B was repeated using the IFD mutant and showed similar immunoblot results as cheA_1_^mut^.

*18. L754-755, tick-mice transmission: “At day 7 after the tick feeding, mice were sacrificed”; L1189, “At day 14 after tick feeding, mice were sacrificed”. Please revise.*

**Response:** Revised as suggested.

*19. L768-773: CheA1IFD mutant was mentioned here. The creation of this mutant in the SI can be moved to L658-663.*

**Response:** Revised as suggested.

*20. Fig. 4, Fig. 5, please add data statistical analysis.*

**Response:** There was no statistically significant difference between WT, mutant, and complement for the results in Figs. 4 and 5.

*21. Fig. 9. Three independent experiments are assumed to be performed. In L1209-1210, qRT-PCR data, “Data from two replicates are expressed as mean fold change relative to WT ± SEM. *, significant difference (P < 0.05).” Please revise.*

**Response:** The immunoblot analysis in Fig. 9B was performed at least three times using independent samples. The qRT-PCR data was repeated twice and the average of two data sets is shown in Fig. 9C.

*22. Fig. 10. Are these data drawn from two independent experiments as indicated in L1219-1220?*

**Response:** Yes, the data shown here are the average of two independent data sets.

*23. Fig S5B. please add data statistical analysis.*

**Response:** This figure has been removed per reviewer #1’s suggestion.

*24. Given that CheW2 interacts with CheA1, the phenotype of the cheW2 mutant should be included in the discussion.*

**Response:** Thank the reviewer for this comment and pertinent discussion has been added as suggested.

*25. It will be very helpful to have a Strains and plasmids list Table or at least indicate the strains used in the Figure legends (e.g., tick-mice infection experiments), as the cheA1 mutation has been created using two different plasmids and in two different strains.*

**Response:** The two *cheA_1_* mutants are named differently, one as *cheA_1_^mut^* and another as *cheA_1_^IFD^*. The figure legend has been updated to reflect the different strains used. Material and Methods have also been updated to clarify the different strains used and their purpose.

**Reviewer #3:**

No concerns

**Part II – Major Issues: Key Experiments Required for Acceptance**

**Reviewer #1:** None

**Reviewer #2:** None

**Reviewer #3:** *Fig. 11: You should compare the data from WT-infected to cheA1 mutant infected, instead of both separately to mock infected. Some clarification on what is being represented in Fig. 11A, that is, are these KEGG pathways upreg, downreg or some up and some down.*

**Response:** Thank the reviewer for this thoughtful suggestion. Fig. 11B has been replotted as suggested. Fig. 11A shows all the statistically differentially expressed genes located in a given pathway, both up and down regulated.

**Part III – Minor Issues: Editorial and Data Presentation Modifications**

**Reviewer #1:** *While the data are strongly suggestive that CheA1 (and for that matter CheY2) are involved in tick transmission, one can argue that a definitive statement to this effect requires determining if spirochetes reach the tick feeding site. The authors are encouraged to perform this experiment.*

**Response:** Technically, this experiment is challenging. We are planning to do this experiment in the future with collaborators who have expertise in tracking and monitoring *B. burgdorferi* cells in ticks using intravital or confocal imaging techniques.

**Reviewer #2:** Please see the summary

**Reviewer #3:**

*Lines 110-111 – “,” around “as well as our group”*

**Response:** Revised as suggested.

*Line 113 – comma after CheA1*

**Response:** Revised as suggested.

*Line 116 – omit the second “the”*

**Response:** Revised accordingly.

*Line 122 – I think you mean proteins not promoters*

**Response:** Agreed and revised as suggested.

*I would omit the paragraph from 154 to 168, except for the part about Borreliaceae (162 – 166) as it distracts from the focus on Borrelia.*

**Response:** Line 154-168 discussed on the significance of the extended P3 domain of CheA and how it is a conserved feature in spirochetes with two CheA isoforms, where the extended P3 domain is a unique feature in chemotaxis-related CheA not only in *Borrelia* but in other spirochetes too.

*Line 172- strains, not strain. Please describe pGA1kan at least briefly. Which strain is being used in fig 2, bbe02 KO or the other?*

**Response:** We have added a brief description for pGA1kan as well as clarify the different strains used in the manuscript in the Material and Methods section.

*Lines 177 “immunoblots using anti-CheA1 antibodies and anti-DnaK antibodies as a control.”*

**Response:** Revised as suggested.

*Line 190 – “chemotaxis-related”*

**Response:** This data has been removed per reviewer #1’s suggestion.

*Line 192 “are” not “is”*

**Response:** This data has been removed per reviewer #1’s suggestion.

*Omit or move to the discussion sentences in lines 191 -195.*

**Response:** This data has been removed per reviewer #1’s suggestion.

*Line 198 – what are “quantitative immunoblots”? I don’t see any description in the materials and methods. I don’t think you need to make claims that might raise concerns about the validity of immunoblot quantitation, just state the levels of CheA1 didn’t change.*

**Response:** This data has been removed per reviewer #1’s suggestion.

*Line 219-220 – repeat, omit.*

**Response:** Line 219-220 was referring to CheA_2_-GFP while line 215-217 was referring to CheA_1_-GFP.

*I am not sure what you mean by “temporally-regulate” lines 204 and 232. I think growth phase and temperature-regulated should be considered, in addition to spatially. Seems like there is complex regulation of CheA1 localization.*

**Response:** Temporal regulation refers to the fact that we observed the localization of CheA_1_ changes from log to stationary phase. We agree with the reviewer that there is complex regulation on the localization of CheA_1_.

*Line 244 – “wild-type”*

**Response:** Revised as suggested.

*Lines 246-247 – Are your measurements (error) really precise to 0.1 nm? I think you need to adjust your significant figures.*

**Response:** The cell velocity was calculated using a software. Revision has been made as suggested.

*Lines 253-255 – Put the last part of the sentence first “A previously developed…was used to determine if CheA1….”*

**Response:** Revised accordingly.

*In the legend for table 1 please define CheA1IFD*

**Response:** Revision has been made accordingly.

*Lines 276 and 318 – “105 cells” and line 277 “were”*

**Response:** Changed as suggested.

*Lines 278 and 294 – did you use qRT-PCR and not qPCR to quantify Bb loads in tissues?*

**Response:** Yes, we used qRT-PCR instead of qPCR to quantify *Bb* burden in mouse tissues. Total RNA was extracted and cDNA was used for quantification.

*Line 329 – “The tail-vein…*

**Response:** Revised as suggested.

*Line 349 – I would say “is likely” instead of “can be”, as you have used human serum for in vitro survival assays and the in vivo studies were done in mice.*

**Response:** Agreed and revised accordingly.

*Lines 353 – “strains”*

**Response:** Changed as suggested.

*Line 355 – I think you mean qPCR, not qRT-PCR.*

**Response:** We used qRT-PCR not qPCR. Total RNA was extracted and cDNA was used for quantification.

*Line 356-358 – Just say that it was not statistically different from WT or comp, as what you have shown is that it actually is not slightly lower.*

**Response:** Agreed and revised accordingly.

*Line 370 – I would not call it an artificial mouse infection, but rather needle inoculation of mice.
I have concerns about figure 8B. It seems like the cheA1com strain does not really complement the phenotype. How do you get error bars for the comp strain in the skin and heart if only one mouse had detectable flaB transcripts? Instead of quantification of flaB just present what tissues had detectable flaB transcripts, quantification doesn’t seem appropriate.*

**Response:** Revision has been made accordingly. For the complement strain error bars, 1 mouse was positive with two other samples having zero copy of *flaB.* We plotted the average of all three readings to get the means. The y-axis is in log scale hence the error bars looked smaller than when plotted in a linear range.

*Line 383 – “the flaB gene”*

**Response:** Changed as suggested.

*Lines 385-388 – This sentence seems to contradict what was stated above and I’m not sure what this means. Also, the data in Fig. 8C seem suspect. I can’t tell how many nymphs were analyzed for the CheA1 mutant but it doesn’t seem possible that the mean could be where it is graphed based on all the data points. Also, the SEM must be larger than represented as numerous nymphs seem to have zero flaB copies.*

**Response:** Eight ticks were analyzed from WT-infected mice and 12 ticks were analyzed from *cheA_1_^mut^* infected mice with three ticks having zero *flaB* copies. The range of the y-axis has been adjusted to better reflect the result (see below figure for the detail).

*Lines 402 and 407-408 – you state that the level of rpoS transcript was not significantly reduced but Fig. 9C shows that it is significant. Please correct the text.*

**Response:** A new Fig. 9C has been added to clarify the statistical significance.

*Please explain how the experiment in Fig. 9D was done in the results section (i.e., cell density, how translation arrested). Also, if protein synthesis was arrested, how does the level of DnaK increase a lot? I am not sure you have arrested protein synthesis. Also, why was the CheA1 comp not done?*

**Response:** Protein turnover was performed as described in the Material and Methods. Stationary phase cells ~10^8^ cells/ml were used. Protein synthesis was arrested with the addition of spectinomycin (100 µg/ml). Cells were harvested at the specified time points for SDS-PAGE. At later time points, we loaded more samples in order to get comparable level of DnaK level as compared to the earlier time points. The level of RpoS can be seen to reduce over time as compared to DnaK in cheA_1_^mut^. Protein turnover on *cheA_1_^com^* was performed and our results showed that the stability of RpoS was restored (Figure S7).

*Line 428 – omit “are”*

**Response:** Deleted as suggested.

*Line 429 – “suggest”, data is plural.*

**Response:** Revised accordingly.

*Lines 442-443 – 107 cells …were.*

**Response:** Changed as suggested.

*Line 460 – Add “Hk2” to the Rrp2-RpoN…*

**Response:** Revised as suggested.

*Line 463 – profiles*

**Response:** Changed as suggested.

*Line 497 – operons*

**Response:** Revision has been made accordingly.

*Line 530 – the localization of CheA1 is not unparalleled as you just pointed out that ParB also has a similar localization, so I would omit this.*

**Response:** Omitted as suggested.

*Line 541 – findings*

**Response:** Changed accordingly.

*Line 570 – mice not mouse*

**Response:** Changed as suggested.

*Line 638 – omit chemotaxis as you have shown CheA1 does not function in chemotaxis (at least in your experiments) and just say “pathogenesis in vivo.”*

**Response:** Revised as suggested.

*Line 743 – what is the source of your “normal human serum”?*

**Response:** Normal human serum was purchased from BioIVT. Material and Methods section has been updated to reflect this.

*In Fig. S7 why do so many Bb seem to not express GFP whether by itself or added to CheA1 or CheA2 (A-C)?*

**Response:** The images were taken using live cells and they were on different planes of focus. Cells that are not in focus under GFP filter will appear having faint or no GFP signals.

*I have a few issues with Fig. 3B legend: Time-lapse implies the same cells were imaged. Use “time course”. It does seem to appear that the puncta are increasing, but not in all cells and quantification might be important. Finally, how do you know the puncta coincide with zones of new peptidoglycan formation?*

**Response:** Revision has been made as suggested. We have removed the statement on zone of new peptidoglycan formation.

*Fig. 4. Line 1153 – I don’t see any numbers for the measurement of diameter of the rings. It would be good to include these data.*

**Response:** Revision has been made accordingly. A new figure has been added to Fig. 4D.

*Were statistical analyses done for Figs. 4 and 5?*

**Response:** Yes, were done but there were no statistically significant difference between WT, cheA_1_^mut^ and cheA_1_^com^.

*Please explain cells were determined to be alive or dead in the serum assay. If a cell is non-motile but no obvious morphological change, is it considered to be viable or not?*

**Response:** If a cell is non-motile with no obvious morphological change, we considered it as non-viable. The same criteria were applied to all three strains during cell counting. This scoring criteria is commonly used in the serum killing assay of *Borrelia* because motility is one of the key factors in determining cell viability^11-14^.

*It would be nice if the murine transcripts DE (Fig. 11) when comparing WT to CheA1 mut by a new volcano plot were verified by qRT-PCR, but not necessary.*

**Response:** We thank the reviewer for this suggestion. We replotted Fig. 11 accordingly.

1	Sze, C. W. & Li, C. Chemotaxis Coupling Protein CheW(2) Is Not Required for the Chemotaxis but Contributes to the Full Pathogenicity of Borreliella burgdorferi. *Infect Immun* **91**, e0000823, doi:10.1128/iai.00008-23 (2023).

2	Sze, C. W., Zhang, K., Kariu, T., Pal, U. & Li, C. Borrelia burgdorferi needs chemotaxis to establish infection in mammals and to accomplish its enzootic cycle. *Infect Immun* **80**, 2485-2492, doi:10.1128/IAI.00145-12 (2012).

3	Dunham-Ems, S. M., Caimano, M. J., Eggers, C. H. & Radolf, J. D. Borrelia burgdorferi requires the alternative sigma factor RpoS for dissemination within the vector during tick-to-mammal transmission. *PLoS Pathog* **8**, e1002532, doi:10.1371/journal.ppat.1002532 (2012).

4	Kumar, D. *et al.* Intravital Imaging of Vascular Transmigration by the Lyme Spirochete: Requirement for the Integrin Binding Residues of the B. burgdorferi P66 Protein. *PLoS Pathog* **11**, e1005333, doi:10.1371/journal.ppat.1005333 (2015).

5	Norman, M. U. *et al.* Molecular mechanisms involved in vascular interactions of the Lyme disease pathogen in a living host. *PLoS Pathog* **4**, e1000169, doi:10.1371/journal.ppat.1000169 (2008).

6	Motaleb, M. A., Miller, M. R., Li, C. & Charon, N. W. Phosphorylation assays of chemotaxis two-component system proteins in Borrelia burgdorferi. *Methods Enzymol* **422**, 438-447, doi:10.1016/S0076-6879(06)22022-6 (2007).

7	Novak, E. A. *et al.* The Borrelia burgdorferi CheY3 response regulator is essential for chemotaxis and completion of its natural infection cycle. *Cell Microbiol* **18**, 1782-1799, doi:10.1111/cmi.12617 (2016).

8	Xu, H. *et al.* Borrelia burgdorferi CheY2 Is Dispensable for Chemotaxis or Motility but Crucial for the Infectious Life Cycle of the Spirochete. *Infect Immun* **85**, doi:10.1128/IAI.00264-16 (2017).

9	Li, C. *et al.* Asymmetrical flagellar rotation in Borrelia burgdorferi nonchemotactic mutants. *Proc Natl Acad Sci U S A* **99**, 6169-6174, doi:10.1073/pnas.092010499 (2002).

10	Motaleb, M. A. *et al.* Borrelia burgdorferi periplasmic flagella have both skeletal and motility functions. *Proc Natl Acad Sci U S A* **97**, 10899-10904, doi:10.1073/pnas.200221797 (2000).

11	Garcia, B. L., Zhi, H., Wager, B., Hook, M. & Skare, J. T. Borrelia burgdorferi BBK32 Inhibits the Classical Pathway by Blocking Activation of the C1 Complement Complex. *PLoS Pathog* **12**, e1005404, doi:10.1371/journal.ppat.1005404 (2016).

12	Kurtenbach, K., Sewell, H. S., Ogden, N. H., Randolph, S. E. & Nuttall, P. A. Serum complement sensitivity as a key factor in Lyme disease ecology. *Infect Immun* **66**, 1248-1251, doi:10.1128/IAI.66.3.1248-1251.1998 (1998).

13	van Dam, A. P. *et al.* Complement-mediated serum sensitivity among spirochetes that cause Lyme disease. *Infect Immun* **65**, 1228-1236, doi:10.1128/iai.65.4.1228-1236.1997 (1997).

14	Lovrich, S. D., Callister, S. M., Schmitz, J. L., Alder, J. D. & Schell, R. F. Borreliacidal activity of sera from hamsters infected with the Lyme disease spirochete. *Infect Immun* **59**, 2522-2528, doi:10.1128/iai.59.8.2522-2528.1991 (1991).
